# Psychomotor Performance in Esports Players: A Comparative Study of Visual Reaction Time and Hand–Eye Coordination

**DOI:** 10.3390/brainsci16090931

**Published:** 2026-08-31

**Authors:** Dan Alexandru Szabo, Adina Stoian, Elena Gabriela Strete, Mircea Stoian, Cristina Veres, Heidrun Adumitrachioaie, Alexandru Rareș Puni, Denisa Elena Hăşmăşan, Dionisie Vladimir Turcu, Gabriel Alexandru Petrovici, Ioan Sabin Sopa

**Affiliations:** 1 Department of Human Movement Sciences, George Emil Palade University of Medicine, Pharmacy, Science, and Technology of Targu Mures, 540139 Targu Mures, Romania; dan-alexandru.szabo@umfst.ro; 2 Department of Pathophysiology, George Emil Palade University of Medicine, Pharmacy, Science, and Technology of Targu Mures, 540139 Targu Mures, Romania; adina.stoian@umfst.ro; 3 Department of Psychiatry, George Emil Palade University of Medicine, Pharmacy, Science, and Technology of Targu Mures, 540142 Targu Mures, Romania; elena.buicu@umfst.ro; 4 Department of Anesthesiology and Intensive Care Medicine, George Emil Palade University of Medicine, Pharmacy, Science, and Technology of Targu Mures, 540142 Targu Mures, Romania; mircea.stoian@umfst.ro; 5 Department of Industrial Engineering and Management, George Emil Palade University of Medicine, Pharmacy, Science, and Technology of Targu Mures, 540088 Targu Mures, Romania; 6 Department of Pediatrics, George Emil Palade University of Medicine, Pharmacy, Science, and Technology of Targu Mures, 540136 Targu Mures, Romania; heidrun.adumitrachioaiei@umfst.ro; 7 Faculty of Physical Education and Sports, “Alexandru Ioan Cuza” University of Iași, 507184 Iași, Romania; alexandru.puni@uaic.ro; 8 Department of Environmental Sciences, Physics, Physical Education and Sports, Lucian Blaga University of Sibiu, 550012 Sibiu, Romania; denisaelena.hasmasan@ulbsibiu.ro (D.E.H.); dionisie.turcu@ulbsibiu.ro (D.V.T.); sabin.sopa@ulbsibiu.ro (I.S.S.); 9 Department of Physical Education and Sport, Faculty of Law and Social Sciences, University 1 December 1918 Alba Iulia, 510009 Alba Iulia, Romania; gabriel.petrovici@uab.ro

**Keywords:** esports, league of legends, psychomotor performance, visual reaction time, hand–eye coordination

## Abstract

**Highlights:**

**What are the main findings?**

- Regular League of Legends players demonstrated faster bilateral visual reaction times, quicker target selection, and shorter response times than control participants.
- Players also showed higher HECOOR coordination, UPDA-SHIF synchronization, and bilateral hand–eye coordination scores, whereas Simon Task accuracy and DIAT-SHIF simultaneity did not differ significantly.

**What are the implications of the main findings?**

- Regular League of Legends participation is associated with a specific psychomotor profile focused on response speed, visual target processing, and coordinated manual performance.
- Esports assessment and training should combine bilateral reaction time, target selection, synchronization, and hand–eye coordination tests instead of relying on a single performance measure.

**Abstract:**

**Background:** This study aimed to compare psychomotor performance between regular League of Legends players and control participants. **Methods:** This cross-sectional study included 71 adults aged 18 to 30 years, divided into a player group, n = 35, and a control group, n = 36. Eleven outcomes assessing visual reaction time, target acquisition, response speed, accuracy, synchronization, and bilateral hand–eye coordination were evaluated using browser-based tasks and CogniFit assessments. Between-group differences were examined using Welch’s *t*-test with Holm adjustment for multiple comparisons. Mann–Whitney tests were conducted as sensitivity analyses. **Results:** After making the adjustments, nine outcomes remained significant. Players showed faster visual reaction times using their right hand (mean difference = −45.33 ms, 95% CI [−60.55, −30.12], d = −1.41) and left hand (−47.67 ms, [−65.09, −30.25], d = −1.29), quicker target acquisition with the right hand (−116.68 ms per target, [−153.69, −79.67], d = −1.49) and left hand (−156.01 ms per target, [−227.35, −84.67], d = −1.03), and shorter response times on the Simon Task (−0.107 s, [−0.153, −0.062], d = −1.12). They also obtained higher scores on HECOOR (1.11%, [0.28, 1.94], d = 0.63), UPDA-SHIF (0.44%, [0.13, 0.76], d = 0.66), and bilateral hand–eye coordination. **Conclusions:** These results indicate that League of Legends players exhibit better psychomotor skills on tasks requiring quick visual responses and good hand–eye coordination. Still, because this study used a cross-sectional design, we cannot draw causal conclusions. More research is needed to confirm these specific effects.

## 1. Introduction

League of Legends is a multiplayer online battle arena game where players make quick decisions, keep a close eye on the action, and use both the mouse and keyboard to control their characters [1,2,3].

Electronic sports, commonly referred to as esports, have developed into organized competitive activities governed by formal rules, ranking systems, and tournament structures. Successful performance requires players to process visual information, maintain attention, make rapid decisions, plan actions, and execute precise motor responses. These demands have increased scientific interest in esports as a context for examining cognitive expertise and perceptual–motor performance [1,2,3].

Esports encompass several game genres, each of which imposes specific demands on players. First-person shooter games emphasize target detection, aiming accuracy, and rapid responses. Sports simulations rely heavily on anticipation and timing. Multiplayer online battle arena games require players to monitor several sources of information, manage resources, communicate with teammates, and select appropriate actions under time pressure. Cognitive and psychomotor performance should therefore be examined in relation to the characteristics of the specific game rather than treating gaming experience as a uniform activity [4,5].

League of Legends is a multiplayer online battle arena game that combines strategic decision-making with repeated keyboard and mouse actions. During a match, players monitor opponents, teammates, moving targets, available resources, ability cooldowns, and information presented on the minimap. They must interpret these stimuli, select an appropriate response, and translate that decision into a precise motor action. Performance depends on the continuous interaction among attention, visual processing, decision-making, reaction speed, and motor coordination [6,7,8].

Psychomotor performance reflects the relationship between cognitive processing and voluntary motor action. It includes stimulus detection, response selection, movement initiation, coordination, speed, and accuracy. In esports, these processes operate through the interaction between the player and digital input devices, particularly the mouse and keyboard. Reaction time indicates how quickly a player detects and responds to a stimulus. Hand–eye coordination describes the ability to guide manual actions using visual information. Both abilities are relevant when players must respond to several changing stimuli within a limited period [9].

Previous research has identified differences between high-frequency League of Legends players and individuals with less gaming experience in selected cognitive and perceptual–motor outcomes. However, these differences have not been observed consistently across all tasks. Ziv et al. examined reaction time and working memory in gamers and non-gamers and showed that participant expectations and testing procedures could influence the observed results [10]. These findings underline the need to interpret gaming-related advantages in relation to the study design, participant characteristics, and selected assessment tasks.

Research focused specifically on League of Legends has reported differences in visuospatial working memory, attentional control, cognitive flexibility, and decision-making. Valls-Serrano et al. found that expert players achieved better visuospatial working memory results than regular players and non-players. Both expert and regular players also responded faster than non-players in an attentional task. However, no significant differences were observed for all executive function measures [7]. In a related study, cognitive flexibility and decision-making performance contributed to the prediction of League of Legends expertise [6]. These findings suggest that gaming experience may be associated with a specific performance profile rather than with a general improvement across all cognitive abilities.

This distinction is important because competitive gaming requires players to balance response speed and accuracy. A faster response does not necessarily represent better performance when it is accompanied by more errors. Players may adopt different response strategies depending on their gaming experience, competitive level, fatigue, motivation, and task demands. Reaction time should therefore be considered together with response accuracy and motor control [11,12,13].

The level and type of gaming expertise may also influence the magnitude of the observed differences. A recent meta-analysis found that esports experts generally performed better than less-experienced participants across several cognitive domains. The most consistent differences involved spatial cognition, attention, and information processing. However, the results varied by game genre, assessment method, and criteria used to define expertise [3]. These findings support the use of game-specific samples and multiple complementary assessment tasks.

The relationship between visual attention and manual movement is particularly relevant in esports. Competitive players repeatedly perform cursor movements, keyboard actions, and rapid responses to visual events. Gao et al. reported better visual reaction time and hand–eye coordination among esports participants than among non-participants. The results also varied by gaming duration and game genre [14]. Because that study included broad esports categories, its findings cannot fully explain the psychomotor characteristics specific to League of Legends.

Intervention research indicates that selected cognitive and motor abilities may change following structured training. Lachowicz et al. reported improvements in reaction time, motor time, and hand–eye coordination after short-term virtual reality training in amateur esports athletes [15]. A related pilot study identified improvements in concentration and alternating attention following a similar intervention [16]. These findings support the adaptability of selected abilities. However, virtual reality training differs from the mouse-and-keyboard interaction used in League of Legends play.

The association between gaming experience and cognitive performance remains complex. Some studies have reported faster information processing, better attentional performance, or stronger working memory among experienced players. Other studies have identified small, task-dependent, or non-significant differences. This variation may reflect differences in participant selection, gaming genre, competitive level, testing conditions, and assessment instruments.

Definitions of regular gaming and esports expertise also vary across studies. Researchers may classify participants according to weekly playing time, competitive rank, years of experience, professional status, or number of ranked matches. Weekly gaming time alone provides an incomplete measure of expertise because players with similar playing durations may compete at different levels. Timokhov et al. found that gaming history and the number of ranked matches predicted League of Legends rank more effectively than general cognitive styles [8]. The characteristics of gaming exposure must therefore be described clearly when comparing players with control participants.

Researchers have also emphasized the need for protocols that combine perceptual, cognitive, and motor measures. Esports coaches consider attention, reaction time, and timing important components of player performance. However, agreement regarding the most appropriate assessment methods remains limited [2,9]. Cyma-Wejchenig et al. demonstrated the value of an esports-specific reaction test when comparing non-professional players with control participants [17]. A protocol containing several complementary tasks may provide a more informative psychomotor profile than a single general reaction-time test.

Digital assessment platforms allow researchers to measure visual reaction time, target selection, response inhibition, divided attention, synchronization, and hand–eye coordination in a single testing session. However, browser-based outcomes may be influenced by computer performance, monitor refresh rate, input latency, mouse characteristics, and familiarity with digital tests. These factors should be considered when interpreting performance differences, particularly for outcomes measured in milliseconds.

Several limitations remain in the available evidence. Many studies combine players from different esports genres, which limits the generalizability of conclusions about League of Legends’ specific demands. Other studies focus primarily on executive functions, working memory, or attention, while bilateral manual reaction speed and hand–eye coordination receive less attention. Research also frequently relies on a single cognitive task or reaction-time outcome. Such an approach cannot describe the broader relationship between response speed, accuracy, synchronization, and motor control. Evidence obtained from Romanian League of Legends players also remains limited.

The present study addresses these gaps through a game-specific and multidimensional assessment of regular League of Legends players. Seven computer-based tests generated 11 outcomes covering visual reaction time, target acquisition, response speed and accuracy, divided attention, visual-motor synchronization, and bilateral hand–eye coordination. The use of several measures within the same protocol allowed speed-based and accuracy-based performance to be examined separately.

This study reports bilateral results in terms of right- and left-hand performance rather than dominant- and non-dominant-hand performance, since the available data were collected based on the hand used during testing, not on functional dominance. It is important to make this distinction in the case of League of Legends, as in this game one hand is generally used to control the mouse and the other to operate the keyboard, and these roles do not always correspond to individuals’ reported handedness. Hence, referring to the hands as right or left provides a more accurate account of the measured tasks.

In a broader sense, the abilities involved in reaction time and hand–eye coordination are not specific to esports; they are also important in team and individual sports such as football, handball, basketball, and tennis, since in these sports athletes have to quickly detect visual stimuli, choose their responses, and coordinate their movements under time pressure [18,19]. It has been found that trained athletes generally perform better than non-athletes in reaction time and visuomotor coordination, although the pattern of performance may vary by sport type, sex, and level of training [20]. These findings indicate that it is relevant to study psychomotor performance in esports and to compare it with other activities that are both physically and cognitively demanding.

The aim of this study was to compare selected psychomotor outcomes between regular League of Legends players and individuals with limited or no regular gaming activity. We hypothesized that regular players would demonstrate faster bilateral visual reaction times, faster bilateral target acquisition, shorter Simon Task response times, and higher coordination and synchronization scores. We also expected regular players to obtain higher bilateral hand–eye coordination scores. Because the study used a cross-sectional design, the findings were interpreted as associations rather than evidence of causal effects.

## 2. Materials and Methods

### 2.1. Study Design

A cross-sectional comparative study was conducted to examine differences in psychomotor performance between regular League of Legends players and individuals with limited or no regular gaming activity. Gaming status was the grouping variable. Seven computer-based tasks generated 11 outcomes covering simple visual reaction time, target acquisition, response speed and accuracy, visuomotor coordination, simultaneity, synchronization, and bilateral hand–eye coordination. Both groups completed the same assessment sequence. Because exposure and outcomes were measured at the same time, the study was designed to identify between-group associations and not causal effects of gaming.

### 2.2. Participants

The final sample comprised 71 Romanian adults aged 18 to 30 years. The League of Legends group included 35 regular players, 31 men and 4 women, with a mean age of 23.40 ± 2.82 years. The control group included 36 participants, 32 men and 4 women, with a mean age of 22.53 ± 2.43 years. The groups did not differ significantly in age, Welch’s t(66) = 1.39, *p* = 0.168, mean difference = 0.87 years, 95% CI [−0.38, 2.12].

Handedness was obtained by means of self-report and not by using a formal, standardized handedness inventory. In League of Legends, the two hands usually perform different functions: one hand generally handles the mouse while the other handles the keyboard. However, the hand that a player uses to control the mouse or the keyboard may differ from person to person and need not match their self-reported functional dominance. For this reason, the bilateral results in the current study were categorized based on the anatomical hand used during testing—that is, either the right or the left hand—rather than by performance with the dominant and non-dominant hand. This method is in line with the available data structure and prevents the implication that formal functional dominance had been assessed. Among the control participants with limited or no regular gaming activity, 33 were right-handed, and 3 were left-handed. The database contained complete demographic and outcome data for all 71 participants.

### 2.3. Eligibility Criteria

Participants in both groups were required to be 18 to 30 years old, understand the study procedures, and provide written informed consent. Individuals were eligible only if they reported no neurological, uncorrected visual, or motor condition that could materially affect visual processing, reaction time, mouse use, or hand–eye coordination. Participants who did not complete the full protocol or did not provide consent were excluded.

Allocation was based on self-reported gaming behavior. For the purposes of this study, “regular, high-frequency League of Legends players” were operationally defined as participants who had at least one year of experience with League of Legends and reported playing the game for at least 20 h per week. The control group comprised individuals who did not play video games regularly or reported fewer than 10 h of total video gaming per week. Individuals reporting 10–19 h of gaming per week did not meet the operational definition of either group and were not included. This classification describes only the amount and duration of gaming exposure. Because competitive rank, tournament participation, and objective ranked-match history were not recorded, participants were not classified as professional, expert, elite, or competitive high-frequency players.

### 2.4. Recruitment and Data Collection

Participants were recruited through social media and online gaming communities. Eligibility was assessed based on age, health status, gaming experience, and weekly gaming time. Before testing, participants completed a demographic questionnaire that recorded age, sex, handedness, gaming status, years of gaming experience, and weekly gaming time.

The control group came from the same general population as the League of Legends players and was recruited through similar channels. This method helped reduce differences between groups in background and recruitment source. While the study did not match participants on every possible confounder, the recruitment strategy aimed to ensure both groups were from a similar local area.

Each participant completed the seven computer-based tasks individually and in the same fixed order: Human Benchmark Reaction Time, Human Benchmark Aim Trainer, CogniFit Simon Task, CogniFit HECOOR, CogniFit DIAT-SHIF, CogniFit UPDA-SHIF, and ARealMe Hand–Eye Coordination. Standard task instructions were presented before each assessment. Participants were instructed to respond as quickly and accurately as possible. Tests with bilateral outcomes were completed separately with the right and left hands, and the platform-recorded scores were entered into the study database.

The retained study records did not document the computer model, operating system, monitor refresh rate, mouse polling rate, browser version, display size, internet latency, room conditions, time of testing, or control of sleep, fatigue, caffeine intake, physical activity, and visual correction. These factors were therefore not used as covariates. Because several tasks ran in a web browser, millisecond-level outcomes may include latency from hardware, the browser, and the input device.

### 2.5. Assessment Instruments

#### 2.5.1. Human Benchmark Reaction Time Test

Simple visual reaction time was assessed using the Human Benchmark Reaction Time Test (https://humanbenchmark.com/tests/reactiontime, accessed on 28 July 2026). A red screen changed to green after a variable delay, and the participant clicked as soon as the green stimulus appeared. The task was performed five times with the right hand and five times with the left hand. The platform calculated the mean latency for each hand in milliseconds. Lower values indicated faster responses. The right-hand and left-hand means were retained as separate outcomes.

#### 2.5.2. Human Benchmark Aim Trainer

Target acquisition and mouse-controlled pointing were assessed using Human Benchmark Aim Trainer (https://humanbenchmark.com/tests/aim, accessed on 28 July 2026). Thirty circular targets appeared successively at different screen positions. The participant moved the cursor to each target and clicked it as rapidly as possible. The task was completed separately with the right and left hands. The platform reported the mean time per target in milliseconds. Lower values indicated faster target acquisition and cursor control.

#### 2.5.3. CogniFit Simon Task

Response selection under stimulus-response compatibility demands was assessed with the CogniFit Simon Task (https://www.cognifit.com/battery-of-tests/simon-task, accessed on 28 July 2026). Participants responded to visual stimuli according to the instructions displayed by the application. Two platform-generated outcomes were retained: mean response time (seconds) and response accuracy (%). Lower response times and higher accuracy values indicated better task performance.

#### 2.5.4. CogniFit Coordination Test, HECOOR

Visuomotor coordination was assessed using the CogniFit HECOOR task (https://www.cognifit.com/battery-of-tests/hecoor-test/coordination-test, accessed on 28 July 2026). Participants used the mouse-controlled cursor to track and respond to a moving visual object. The platform-generated coordination score was expressed as a percentage, with higher values indicating greater task accuracy.

#### 2.5.5. CogniFit Simultaneity Test, DIAT-SHIF

The ability to process concurrent task demands was assessed using the CogniFit DIAT-SHIF task (https://www.cognifit.com/battery-of-tests/diat-shif-test/simultaneity-test, accessed on 28 July 2026). Participants responded to simultaneous requirements presented by the application. The retained platform-generated simultaneity score was expressed as a percentage. Higher values indicated better performance.

#### 2.5.6. CogniFit Synchronization Test, UPDA-SHIF

Temporal coordination of a manual response with a moving visual stimulus was assessed using the CogniFit UPDA-SHIF task (https://www.cognifit.com/battery-of-tests/upda-shif-test/synchronization-test, accessed on 28 July 2026). Participants monitored an onscreen stimulus and responded at the required moment. The platform-generated synchronization score was expressed as a percentage, with higher values indicating better performance on the task.

#### 2.5.7. ARealMe Hand–Eye Coordination Test

Bilateral hand–eye coordination was assessed using the ARealMe Hand–Eye Coordination Test (https://www.arealme.com/eye-hand-coordination-test/en/, accessed on 28 July 2026). The test comprised 20 progressively more difficult rounds. In each round, two balls moved along an axis toward a dashed target area, and the participant clicked to stop them within that area. The scoring weights across the 20 rounds summed to 100 points. Stopping both balls within the target area awarded the full score for that round, stopping one ball awarded half of the available score, and stopping neither ball awarded no points. The task was completed separately with the right and left hands. The total score for each hand ranged from 0 to 100 points, with higher values indicating better performance.

The Human Benchmark and ARealMe tools are publicly available browser-based performance tasks rather than diagnostic neuropsychological instruments. Their platform scores were analyzed without transformation. Public technical information on test–retest reliability, measurement error, minimum detectable difference, and the influence of specific hardware configurations was not available for all tasks. Results were therefore interpreted as performance on the administered digital tasks rather than as clinical measurements of latent ability.

### 2.6. Outcome Measures

The primary analysis included 11 outcomes: right-hand and left-hand visual reaction time, measured in milliseconds; right-hand and left-hand Aim Trainer time, measured in milliseconds per target; Simon Task response time, measured in seconds; Simon Task accuracy, expressed in percentage points; HECOOR coordination, expressed in percentage points; DIAT-SHIF simultaneity, expressed in percentage points; UPDA-SHIF synchronization, expressed in percentage points; and right-hand and left-hand ARealMe hand–eye coordination, expressed in points on a scale from 0 to 100.

Lower values represented better performance for visual reaction time, Aim Trainer, and Simon Task response time. Higher values represented better performance for Simon Task accuracy, HECOOR, DIAT-SHIF, UPDA-SHIF, and ARealMe hand–eye coordination. Right-hand and left-hand outcomes were analyzed as distinct measures. They were not relabeled as dominant-hand and non-dominant-hand outcomes.

### 2.7. Data Quality and Statistical Analysis

The database was checked for duplicate records, missing values, impossible values, and inconsistencies between group labels and demographic variables. No missing outcome values or range violations were identified. Descriptive statistics were calculated as the mean and standard deviation for all outcomes. Median and interquartile range were also examined for percentage outcomes because several distributions were concentrated near the upper boundary of 100%. The proportion of participants who reached the maximum score was inspected to identify potential ceiling effects.

Age normality was evaluated separately in each group using the Anderson–Darling test. No significant departure from normality was identified for the League of Legends group (*p* = 0.337) or the control group (*p* = 0.258). Between-group differences in age and the 11 psychomotor outcomes were tested using Welch’s independent-samples *t*-test. Welch’s test was selected because it does not assume equal variances and remains appropriate with slightly unequal group sizes. For each outcome, the analysis reported the Welch t-statistic, adjusted degrees of freedom, raw two-sided *p*-value, mean difference, and 95% confidence interval.

Between-group differences in sex and handedness distributions were examined using Fisher’s exact test because of the small expected frequencies in several cells.

Effect sizes were calculated as Cohen’s d using the pooled standard deviation and were interpreted with attention to both direction and magnitude. To control the familywise error rate across the 11 psychomotor comparisons, raw *p*-values were adjusted with the sequential Holm procedure. Statistical significance was defined as a Holm-adjusted *p*-value below 0.05.

The Mann–Whitney U test was used in a sensitivity analysis because several bounded-accuracy variables exhibited skewness and ceiling effects. The main interpretation required agreement between the Welch estimate and the distribution-free sensitivity analysis, while the Holm-adjusted Welch result defined statistical significance in the primary analysis.

The original inferential analyses were conducted in Minitab version 20.3. Data verification, Holm adjustment, effect-size calculations, medians, interquartile ranges, ceiling frequencies, and Mann–Whitney sensitivity analyses were independently reproduced from the participant-level database using Python, version [3.12.4] (Python Software Foundation, Wilmington, DE, USA), pandas, version [2.2.2] (pandas development team/NumFOCUS, Austin, TX, USA), NumPy, version [2.0.2] (NumPy developers/NumFOCUS, Austin, TX, USA), and SciPy, version [1.13.1] (SciPy developers/NumFOCUS, Austin, TX, USA). All tests were two-sided. No a priori sample-size calculation was documented. Therefore, the analyses should be regarded as exploratory, especially for small effects and outcomes affected by ceiling compression.

### 2.8. Bias Control and Interpretation

Both groups completed the same task sequence and received the same task instructions. Multiplicity was addressed using the Holm adjustment, and bounded outcomes were assessed using a rank-based sensitivity analysis. The study did not randomize exposure, blind participants or assessors, match participants on gaming-related device experience, or control hardware and testing conditions. Sex distribution was similar across groups, but the small number of women prevented sex-stratified analysis. Handedness was recorded, although the database stored bilateral results as right-hand and left-hand scores. These design features were considered when interpreting the findings.

### 2.9. Ethical Considerations

The study was conducted in accordance with the Declaration of Helsinki. The Scientific Research Ethics Committee of the George Emil Palade University of Medicine, Pharmacy, Science, and Technology of Târgu Mureș, Romania, approved the protocol, approval no. 2749 of 26 January 2024. Participation was voluntary. Before enrolment, participants received information about the study purpose, procedures, confidentiality safeguards, and the right to withdraw without consequences. Written informed consent was obtained from every participant before data collection.

## 3. Results

### 3.1. Participant Characteristics

The final analysis included 71 participants. The League of Legends group comprised 35 regular players, while the control group comprised 36 participants. The players had a mean age of 23.40 ± 2.82 years, compared with 22.53 ± 2.43 years in the control group. The difference of 0.87 years was not statistically significant, Welch’s t (66.00) = 1.39, *p* = 0.168, 95% CI [−0.38, 2.12].

The League of Legends group included 31 men and 4 women. The control group included 32 men and 4 women. Sex distribution did not differ between groups (Fisher’s exact test, *p* = 1.000).

Thirty-four players and 33 control participants reported right-hand dominance. One player and three control participants reported left-hand dominance. The difference in handedness distribution was not statistically significant (Fisher’s exact test, *p* = 0.614).

### 3.2. Between-Group Differences in Psychomotor Performance

Table 1 presents the results for the 11 psychomotor outcomes. After Holm adjustment, statistically significant between-group differences remained for nine outcomes. Simon Task accuracy and DIAT-SHIF simultaneity did not differ significantly between groups.

### 3.3. Visual Reaction Time and Target Acquisition

Regular League of Legends players recorded shorter visual reaction times with both hands. For the right hand, players responded 45.33 ms faster than controls (95% CI [−60.55, −30.12]; Holm-adjusted *p* < 0.001; d = −1.41). For the left hand, the difference was 47.67 ms (95% CI [−65.09, −30.25]), Holm-adjusted *p* < 0.001, d = −1.29. Both differences represented large standardized effects.

The players also completed the Aim Trainer task faster with both hands. Right-hand performance was 116.68 ms per target faster among players, 95% CI [−153.69, −79.67], Holm-adjusted *p* < 0.001, d = −1.49. Left-hand performance was 156.01 ms per target faster, 95% CI [−227.35, −84.67], Holm-adjusted *p* < 0.001, d = −1.03.

**Figure 1:**
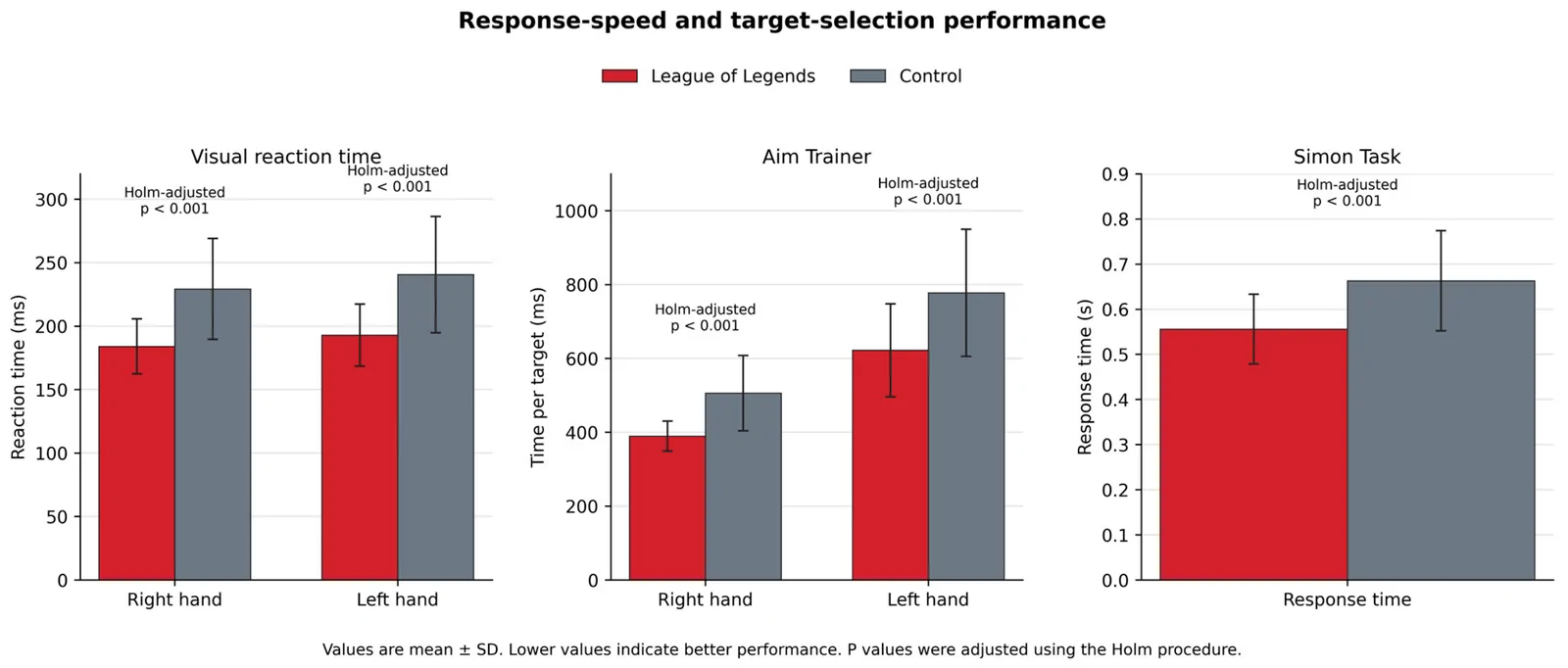
Visual reaction time and target-acquisition performance in regular League of Legends players and control participants.

Figure 1 presents the distributions of bilateral visual reaction time and target-acquisition performance. Regular League of Legends players recorded shorter response times for both hands across the two tasks.

### 3.4. Simon Task Performance

Players recorded a shorter mean response time on the Simon Task than controls, 0.556 ± 0.077 s versus 0.663 ± 0.111 s. The mean difference was −0.107 s, 95% CI [−0.153, −0.062], Holm-adjusted *p* < 0.001, d = −1.12.

Simon Task accuracy was similar between groups. Players achieved 99.23 ± 1.68%, while controls achieved 99.14 ± 1.73%. The difference of 0.09 percentage points was not statistically significant (Holm-adjusted *p* = 0.825; d = 0.05).

### 3.5. Coordination, Simultaneity, and Synchronization

The League of Legends group obtained a higher HECOOR score than the control group, with a mean difference of 1.11 percentage points (95% CI [0.28, 1.94]), Holm-adjusted *p* = 0.042, d = 0.63.

The mean DIAT-SHIF score was 1.67 percentage points higher among players. However, its 95% CI included zero (95% CI [−0.20, 3.54]), and the difference did not remain significant after Holm adjustment (adjusted *p* = 0.157; d = 0.42).

Players also obtained a higher UPDA-SHIF score. The mean difference was 0.44 percentage points, 95% CI [0.13, 0.76], Holm-adjusted *p* = 0.037, d = 0.66. Despite its statistical significance, the absolute difference was small and occurred near the maximum possible score.

### 3.6. Bilateral Hand–Eye Coordination

Players achieved higher ARealMe hand–eye coordination scores for both hands. For the right hand, the difference was 13.14 points (95% CI [6.79, 19.49]), Holm-adjusted *p* < 0.001, d = 0.98. For the left hand, the difference was 6.83 points, 95% CI [1.55, 12.11], Holm-adjusted *p* = 0.042, d = 0.61.

The standardized effect was larger for the right hand. However, the outcomes were classified by the hand used during testing, not by dominant- or non-dominant-hand status.

**Figure 2:**
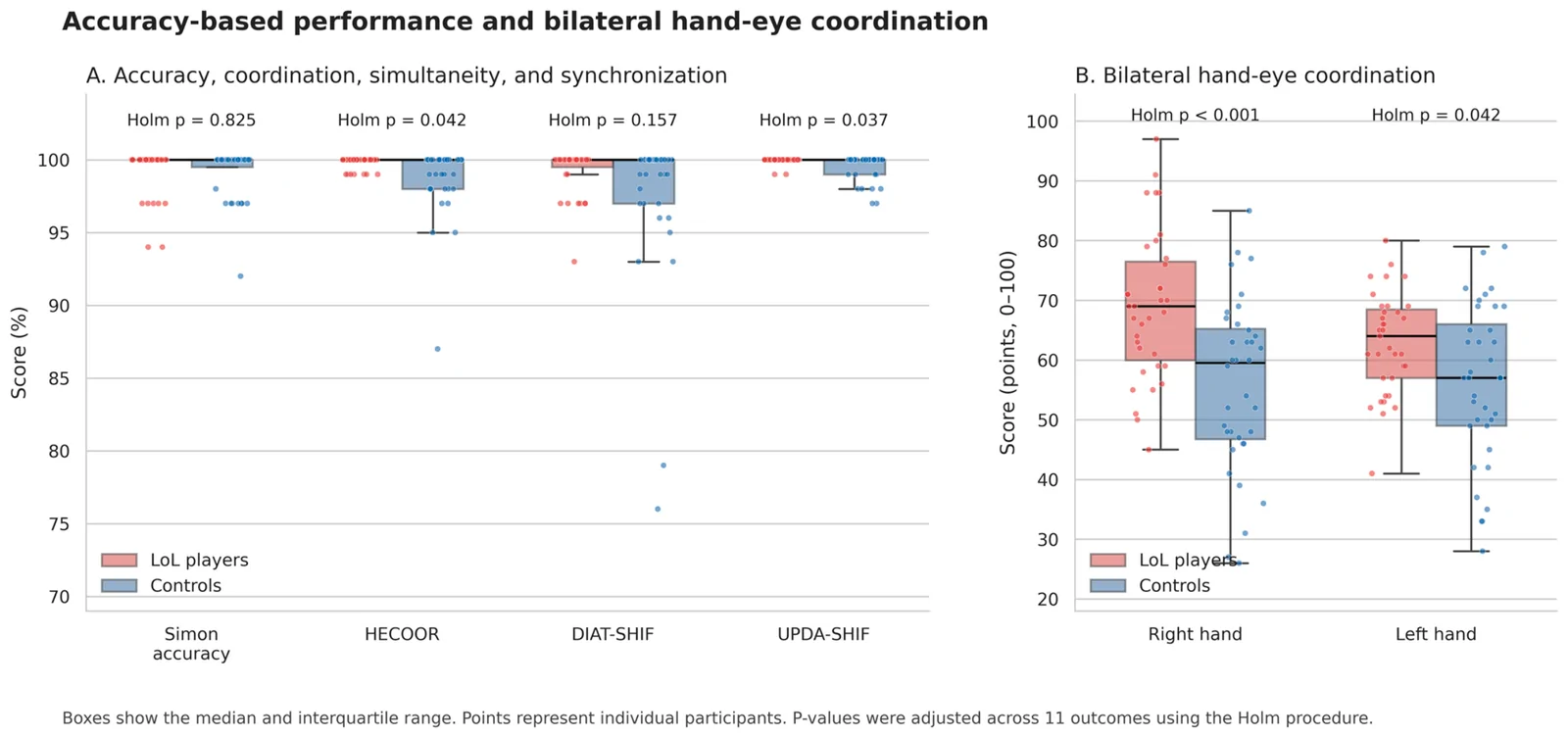
Accuracy-based performance and bilateral hand–eye coordination in regular League of Legends players and control participants. Panel (**A**) presents scores for Simon Task accuracy, HECOOR coordination, DIAT-SHIF simultaneity, and UPDA-SHIF synchronization. Panel (**B**) presents bilateral ARealMe hand–eye coordination scores. Boxes indicate the median and interquartile range, while points represent individual participants. Reported *p*-values were adjusted across 11 comparisons using the Holm procedure.

Figure 2 summarizes the percentage-based CogniFit outcomes and bilateral ARealMe hand–eye coordination scores. The League of Legends group recorded higher mean values for HECOOR and UPDA-SHIF. DIAT-SHIF did not remain statistically significant after Holm adjustment. Bilateral hand–eye coordination scores were higher among players, with a larger difference for the right hand.

### 3.7. Sensitivity Analysis and Ceiling Effects

The Mann–Whitney sensitivity analysis supported the direction and statistical interpretation of the primary results. Significant distributional differences were found for the nine outcomes that remained significant after Holm adjustment in the Welch analysis. Simon Task accuracy remained non-significant (*p* = 0.705), as did DIAT-SHIF (*p* = 0.088).

Several percentage-based outcomes showed substantial ceiling effects. Table 2 presents their median values, interquartile ranges, and maximum-score frequencies.

The concentration of values at 100% reduced these tasks’ ability to distinguish performance at the upper end of their scales. This issue was most pronounced for UPDA-SHIF, where 94.3% of players and 72.2% of controls obtained the maximum score. Therefore, the statistically significant HECOOR and UPDA-SHIF results should be interpreted together with their small absolute differences and restricted score distributions.

## 4. Discussion

The present study compared 11 psychomotor outcomes between regular League of Legends players and control participants with limited or no regular gaming activity. Regular players demonstrated shorter bilateral visual reaction times, faster bilateral target acquisition, shorter Simon Task response times, higher HECOOR coordination and UPDA-SHIF synchronization scores, and better bilateral hand–eye coordination. Nine of the eleven between-group differences remained statistically significant after Holm adjustment. No significant differences were identified for Simon Task accuracy or DIAT-SHIF simultaneity.

The findings suggest that regular League of Legends players have faster psychomotor processing and better visuomotor coordination than non-gaming controls. Their average reaction time was similar to that reported in sports such as fencing and table tennis, which require quick perceptual–motor skills. However, these comparisons should be made carefully, since performance depends on the specific sensorimotor demands of each activity. Therefore, our results do not show an overall advantage in motor performance, but rather a specific benefit in reaction speed and eye–hand coordination.

Overall, these findings support most of the study’s hypotheses and indicate that regular League of Legends participation is associated primarily with response speed, visuomotor control, and coordinated manual performance. However, the cross-sectional design does not establish that gaming experience caused the observed differences.

### 4.1. Visual Reaction Time

The clearest differences were observed for outcomes based on response speed. League of Legends players responded approximately 45 ms faster with the right hand and 48 ms faster with the left hand during the visual reaction time task. The corresponding effect sizes were large, d = −1.41 and d = −1.29. Previous studies have similarly reported faster visual processing and response execution among experienced video game players compared with individuals with limited gaming experience [21,22,23]. Comparable differences have also been observed in high-frequency League of Legends players and across other competitive gaming genres [3,4,14].

This pattern corresponds with several demands of League of Legends. During gameplay, players must detect rapidly changing visual events, identify relevant information, ignore competing stimuli, select an appropriate action, and execute a keyboard or mouse response. Research on visual selective attention suggests that gaming experience may be associated with more efficient attentional allocation and faster processing of task-relevant stimuli [24]. Neurocognitive evidence also indicates that experienced players may differ in the recruitment of attentional control networks [25].

The bilateral nature of the results is particularly relevant. League of Legends distributes manual actions between the two hands. One hand generally controls the mouse, while the other operates the keyboard. Faster responses with both hands may therefore be associated with repeated exposure to tasks requiring continuous bilateral interaction with digital input devices. However, the reaction time test measured simple visual responses rather than game-specific decisions. These outcomes should consequently be interpreted as components of a broader psychomotor profile and not as direct indicators of competitive gaming performance.

### 4.2. Target Acquisition and Cursor Control

The largest standardized difference was observed for right-hand Aim Trainer performance (d = −1.49). Regular players required approximately 117 ms less per target than controls. A large difference was also identified for the left hand, d = −1.03, with players requiring approximately 156 ms less per target.

Aim Trainer involved more than a simple response to stimulus onset. Participants had to detect each target, identify its location, plan and execute the cursor movement, and complete an accurate click. The task therefore combined visual detection, spatial selection, movement planning, cursor control, and response execution.

The larger effect for the right hand may be related to regular mouse use among League of Legends players. Experimental evidence suggests that action video game training may improve visuomotor control and the sensorimotor system’s responsiveness to visual information [26]. Gaming experience may also support the development of more efficient perceptual templates for processing relevant visual stimuli [27]. These mechanisms could partly explain the faster target acquisition recorded in the present study. Nevertheless, mouse familiarity was not measured, and the specific contribution of League of Legends experience cannot be separated from broader computer use.

### 4.3. Simon Task Performance

Regular players also showed shorter Simon Task response times, with a large effect size (d = −1.12). Their mean accuracy was almost identical to that of the control group: 99.23% versus 99.14%. This pattern indicates that the faster responses among players were not accompanied by a statistically detectable reduction in accuracy, although the pronounced ceiling effect limits this interpretation.

Previous studies have associated video game experience with reduced task-switching costs and greater cognitive flexibility [28,29]. Research involving first-person shooter players has also identified advantages in selected components of cognitive control and working memory, although comparable effects were not consistently observed across all inhibition measures [30]. Differences associated with gaming may involve both the selection of relevant stimuli and the subsequent response processes [31].

Research specifically involving League of Legends offers additional context. Higher-ranked players have shown better cognitive flexibility, interference control, and sustained response accuracy than lower-ranked players [32]. Cognitive flexibility and decision-making performance have also been associated with game-specific performance indicators [6]. Visuospatial working memory and attentional control may distinguish expert players, regular players, and non-players, although these differences do not consistently extend to all cognitive outcomes [7].

The absence of a significant difference in Simon Task accuracy requires careful interpretation. Both groups performed close to the maximum possible score. The median was 100% in both groups, while 80.0% of players and 75.0% of controls reached the maximum value. This ceiling effect limited variability and reduced the task’s ability to distinguish participants at the upper end of performance. Therefore, the non-significant result does not demonstrate equivalent response control under more demanding conditions. Future studies could employ more trials, shorter response windows, a greater proportion of incongruent stimuli, or adaptive difficulty.

Research on cognitive performance among gamers has not yielded consistent findings. Reported differences in reaction time and working memory may depend on recruitment methods, participant expectations, testing procedures, gaming genre, weekly gaming activity, and the cognitive outcomes selected [10,33]. Meta-analytic evidence indicates positive associations between action gaming and perception, top-down attention, and spatial cognition, but also shows substantial heterogeneity among studies [34].

A more recent meta-analysis reported larger differences in cross-sectional comparisons than the improvements identified in experimental training studies [35]. This distinction is important for the present findings. Large between-group effects cannot be attributed exclusively to gaming practice. Pre-existing psychomotor abilities, motivation, self-selection, computer experience, and other behavioral factors may also contribute to group membership.

### 4.4. Coordination, Simultaneity, and Synchronization

Regular League of Legends players had higher HECOOR coordination scores, with a moderate effect size (d = 0.63). They also achieved higher UPDA-SHIF synchronization scores (d = 0.66). These findings suggest better performance on tasks that require integrating visual information, manual action, cursor control, and response timing.

League of Legends repeatedly requires players to coordinate manual responses with moving visual elements, character positions, changing targets, and brief action windows. The observed differences are consistent with evidence linking gaming experience to visuomotor control, target tracking, and rapid motor adjustment [23,36]. Relationships between gaming experience and cognitive or motor performance may also vary across gaming genres and control systems [36]. This variation supports the separate examination of specific games instead of treating all forms of video gaming as a single exposure.

The mean DIAT-SHIF score was higher among players, but the difference did not remain statistically significant after Holm adjustment. Its effect size was small to moderate, d = 0.42, and the 95% confidence interval included zero. The groups could therefore not be clearly distinguished through the simultaneity outcome measured by this task.

League of Legends requires players to monitor teammates, opponents, ability cooldowns, objectives, resources, and minimap information. A more pronounced difference in simultaneity might therefore have been expected. However, gaming-related differences depend in part on how closely the assessment task reflects the game’s cognitive demands [37]. A general simultaneity task may not reproduce the visual complexity, time pressure, strategic relevance, and consequences of decisions made during a League of Legends match.

Ceiling effects further complicate the interpretation of these outcomes. Mean HECOOR, DIAT-SHIF, and UPDA-SHIF scores ranged from 97.56% to 99.94%. Maximum scores were recorded by 80.0% of players and 52.8% of controls for HECOOR, by 74.3% and 55.6% for DIAT-SHIF, and by 94.3% and 72.2% for UPDA-SHIF. These restricted distributions may have reduced the tasks’ ability to discriminate between performance levels.

The significant HECOOR and UPDA-SHIF results should therefore be interpreted alongside their absolute differences of only 1.11 and 0.44 percentage points. Statistical significance does not necessarily indicate a large practical difference, particularly when scores cluster near the upper limit. Future investigations should use more demanding visuomotor and divided-attention tasks with a wider effective scoring range.

### 4.5. Bilateral Hand–Eye Coordination

Regular players obtained higher bilateral hand–eye coordination scores. The difference was approximately 13 points for the right hand and 7 points for the left hand. The effect was large for the right hand (d = 0.98) and moderate for the left hand (d = 0.61).

The larger right-hand difference corresponds with the Aim Trainer findings and may reflect the extensive use of mouse-controlled actions during League of Legends play. However, the results were classified by the hand used during testing rather than by dominant-hand status. The findings cannot, therefore, establish whether the greater right-hand difference resulted from hand dominance, mouse use, or both.

These results support the separate assessment of the two hands. A single combined score could conceal differences associated with the distinct functional roles of the mouse and keyboard hands. The findings are also consistent with research indicating that gamers’ cognitive and motor profiles may vary with game demands, gaming genre, and control system [38,39].

Nevertheless, the ARealMe task is a publicly accessible browser-based assessment with limited published information regarding test–retest reliability, measurement error, and sensitivity to hardware characteristics. The results should therefore be interpreted as performance on the administered task, not as a clinical measure of general hand–eye coordination.

### 4.6. Overall Pattern and Practical Implications

The pattern of effect sizes provides information beyond what statistical significance alone can convey. The largest effects were observed for right-hand target acquisition, bilateral visual reaction time, Simon Task response time, and left-hand target acquisition. Moderate effects were identified for HECOOR coordination, UPDA-SHIF synchronization, and left-hand hand–eye coordination. The biggest differences therefore concerned rapid visual detection, target localization, cursor movement, and manual response execution. Differences were smaller for percentage-based accuracy and coordination outcomes, especially when both groups performed near the maximum score.

This pattern is consistent with the three-level model of esports cognitive expertise, which distinguishes basic cognitive abilities, game-specific cognitive skills, and high-level strategic expertise [3]. The present protocol primarily assessed basic psychomotor and cognitive performance. It did not directly evaluate tactical anticipation, communication, game knowledge, strategic decision-making, or team coordination.

The findings may inform a multidimensional assessment of esports. Coaches and researchers could assess reaction speed, target acquisition, bilateral coordination, synchronization, and response accuracy separately. Testing both hands may help identify performance asymmetries that remain hidden when only a general score is used. Relevant training tasks may include visual reaction exercises, cursor-control activities, bilateral coordination drills, and exercises requiring rapid responses without reduced accuracy.

Such measures should complement direct indicators of gaming performance, including competitive rank, preferred role, match history, number of ranked matches, and recent activity. Cognitive abilities and gaming expertise can be examined more accurately when psychomotor assessments are combined with objective tournament and in-game performance data [39]. Gaming history and ranked match participation may predict League of Legends expertise more effectively than general cognitive styles [8]. Weekly playing time alone should therefore not be treated as a complete indicator of gaming expertise.

Intervention studies suggest that selected cognitive and psychomotor abilities may respond to structured digital, cognitive-motor, or virtual-reality training [9,15,16]. However, the magnitude of these effects may depend on training duration, task specificity, initial expertise, and the outcome evaluated. Because the present study did not include an intervention, it cannot determine whether repeated participation in League of Legends produced the observed differences.

Reaction time and visuomotor coordination are important for performance not only in esports but also in open-skill sports like football, basketball, handball, and tennis. In these sports, players need to quickly detect stimuli and respond accurately under time pressure [40,41,42,43,44].

Recent studies indicate that such skills are developed through experience gained in specific sports and through the requirements of each task, particularly in fast-paced situations that demand continuous visual attention and rapid decision-making [42,43,44]. Our results probably reflect adaptations to repeated sensorimotor challenges rather than indicating a general cognitive advantage. This is consistent with other research suggesting that competitive gaming can enhance visual processing and coordinated action in time-limited tasks [44,45,46].

### 4.7. Limitations and Future Research

Several limitations should be considered when interpreting the results. First, the cross-sectional design prevents causal inference. The study cannot determine whether regular League of Legends participation contributed to improved psychomotor performance, or whether individuals with faster responses and stronger visuomotor coordination were more likely to play the game regularly. Meta-analytic evidence showing larger effects in cross-sectional studies than in controlled training studies reinforces this concern [32]. The seven tasks were administered in a fixed order rather than in a counterbalanced or randomised sequence. Consequently, practice, fatigue, and order effects may have influenced performance in the later assessments.

Second, the sample included only 71 Romanian adults aged 18 to 30 years. No a priori sample-size calculation was documented. The findings may therefore have limited statistical precision, particularly for small effects. They cannot be generalized directly to professional high-frequency League of Legends players, adolescents, older adults, or players from other cultural and competitive contexts.

Third, the sample included only eight women. This distribution prevented a meaningful sex-stratified analysis and limits the generalizability of the findings. Future studies should recruit larger, more balanced samples and examine potential interactions among sex, handedness, competitive level, and preferred gaming role.

Fourth, gaming exposure was classified primarily through self-reported weekly activity and experience. Self-reported playing time may be affected by recall error. It also does not capture practice intensity, recent activity, preferred role, match quality, or competitive expertise. Competitive rank was not recorded. The gaming group should consequently be interpreted as a group of regular, high-frequency League of Legends players rather than expert or elite players. Future studies should verify exposure through objective game records and ranked-match histories.

Fifth, the study did not measure general computer use, mouse familiarity, keyboard experience, or previous exposure to browser-based psychomotor tests. Differences in digital device experience could partly explain the advantages observed in tasks requiring cursor movement and clicking.

Sixth, hardware and testing conditions were not fully standardized. Monitor refresh rate, display latency, mouse polling rate, computer configuration, operating system, browser performance, and input device characteristics can influence outcomes measured in milliseconds. Future studies should use the same laboratory equipment and testing environment for all participants.

Seventh, the protocol did not control sleep, fatigue, caffeine intake, recent physical activity, visual correction, motivation, or testing time. These factors can influence reaction time, attention, and manual performance and should be standardized or included as covariates in future investigations.

Eighth, Human Benchmark and ARealMe are publicly accessible browser-based tasks rather than fully validated diagnostic instruments. Published information regarding their reliability, measurement error, sensitivity, and hardware dependence remains limited. Future research should replicate the findings using validated neuropsychological and laboratory-based measures.

Ninth, several percentage-based outcomes showed pronounced ceiling effects. These effects restricted variability and limited the tasks’ ability to discriminate among high-performing participants. More difficult tasks, adaptive testing, additional trials, and participant-level distribution analyses may provide greater sensitivity.

Tenth, the study does not include information on participants’ general computer use, familiarity with the mouse, experience with games other than League of Legends, or prior exposure to browser-based assessment tasks. This is a serious threat to the study’s internal validity, as the differences between the groups might reflect general computer proficiency rather than their participation in League of Legends. It is therefore necessary for subsequent studies to examine and account for these potential confounding factors.

Eleventh, the absence of reporting the duration of each task or confirmation of whether standardized break or familiarization procedures were used before testing was due to the available data not including this information. This makes it harder to reproduce and interpret the protocol, since differences in preparation time or in how participants became accustomed to the tasks may have affected their performance. Also, using a fixed task order could have caused practice, fatigue, or sequence effects. Future studies should clearly report task duration and procedures, and use randomized or counterbalanced task orders to reduce order-related bias.

Finally, the analysis included 11 psychomotor outcomes, increasing the risk of false-positive findings. Holm adjustment controlled the familywise error rate, and nine differences remained significant. The Mann–Whitney sensitivity analysis supported the direction and statistical interpretation of these results. Nevertheless, HECOOR, UPDA-SHIF, and left-hand coordination had adjusted *p*-values close to 0.05 and require independent replication.

## 5. Conclusions

Regular League of Legends players demonstrated faster bilateral visual responses, faster target acquisition, shorter Simon Task response times, and higher performance scores on the ARealMe hand–eye coordination task than control participants with limited or no regular gaming activity. Differences were also identified for HECOOR coordination and UPDA-SHIF synchronization, although these outcomes showed ceiling effects and small absolute between-group differences. Simon Task accuracy and DIAT-SHIF simultaneity did not differ significantly between groups.

These findings indicate an association between regular League of Legends participation and performance on selected browser-based psychomotor tasks. They do not demonstrate that gaming experience produced the observed differences. Longitudinal and experimental studies using standardized hardware, validated assessment instruments, objective gaming records, and more representative samples are required to clarify the direction and practical relevance of these relationships.

Overall, these results show a correlation, not a cause-and-effect relationship, because the study used a cross-sectional design. Still, the improvements in visual reaction time and hand–eye coordination suggest that regularly playing fast-paced esports might help people develop specific sensorimotor skills. Future studies should examine whether these effects also appear in other types of games and sports and whether they lead to broader cognitive-motor benefits when tested in controlled settings.

## Figures and Tables

**Table 1:** Comparison of psychomotor outcomes between regular League of Legends players and controls.

Outcome	Players, Mean ± SD	Controls, Mean ± SD	Mean Difference, 95% CI	Welch’s t(df)	Raw *p*-Value	Holm-Adjusted *p*-Value	Cohen’s d
Visual reaction time, right hand, ms	184.00 ± 21.73	229.33 ± 39.85	−45.33 [−60.55, −30.12]	−5.97 (54.44)	<0.001	<0.001	−1.41
Visual reaction time, left hand, ms	192.89 ± 24.49	240.56 ± 45.83	−47.67 [−65.09, −30.25]	−5.49 (53.80)	<0.001	<0.001	−1.29
Aim Trainer, right hand, ms per target	389.49 ± 40.61	506.17 ± 102.34	−116.68 [−153.69, −79.67]	−6.35 (46.01)	<0.001	<0.001	−1.49
Aim Trainer, left hand, ms per target	621.71 ± 125.83	777.72 ± 172.13	−156.01 [−227.35, −84.67]	−4.37 (64.11)	<0.001	<0.001	−1.03
Simon Task response time, s	0.556 ± 0.077	0.663 ± 0.111	−0.107 [−0.153, −0.062]	−4.74 (62.33)	<0.001	<0.001	−1.12
Simon Task accuracy, %	99.23 ± 1.68	99.14 ± 1.73	0.09 [−0.72, 0.90]	0.22 (69.00)	0.825	0.825	0.05
HECOOR coordination, %	99.80 ± 0.41	98.69 ± 2.42	1.11 [0.28, 1.94]	2.70 (37.02)	0.010	0.042	0.63
DIAT-SHIF simultaneity, %	99.23 ± 1.57	97.56 ± 5.33	1.67 [−0.20, 3.54]	1.81 (41.22)	0.078	0.157	0.42
UPDA-SHIF synchronization, %	99.94 ± 0.24	99.50 ± 0.91	0.44 [0.13, 0.76]	2.82 (39.79)	0.007	0.037	0.66
Hand–eye coordination, right hand, points	69.00 ± 12.36	55.86 ± 14.41	13.14 [6.79, 19.49]	4.13 (67.95)	<0.001	<0.001	0.98
Hand–eye coordination, left hand, points	62.86 ± 8.50	56.03 ± 13.27	6.83 [1.55, 12.11]	2.59 (59.78)	0.012	0.042	0.61

Note: Mean differences and Cohen’s d values were calculated as League of Legends players minus controls. Negative values indicate shorter completion or response times among players. Positive values indicate higher accuracy or coordination scores among players. Lower scores represent better performance for reaction time, Aim Trainer, and Simon Task response time. Higher scores represent better performance for the remaining outcomes.

**Table 2:** Distribution and ceiling effects for bounded percentage outcomes.

Outcome	Players, Median [IQR]	Controls, Median [IQR]	Players at 100%, n (%)	Controls at 100%, n (%)	Mann–Whitney *p*-Value
Simon Task accuracy	100 [100, 100]	100 [99.5, 100]	28 (80.0%)	27 (75.0%)	0.705
HECOOR coordination	100 [100, 100]	100 [98, 100]	28 (80.0%)	19 (52.8%)	0.004
DIAT-SHIF simultaneity	100 [99.5, 100]	100 [97, 100]	26 (74.3%)	20 (55.6%)	0.088
UPDA-SHIF synchronization	100 [100, 100]	100 [99, 100]	33 (94.3%)	26 (72.2%)	0.011

## Data Availability

The data supporting the findings of this study are available from the corresponding author upon reasonable request. The data are not publicly available due to participant privacy and ethical considerations.
